# The spectrum of renal diseases with lupus-like features: a single-center study

**DOI:** 10.1080/0886022X.2022.2057862

**Published:** 2022-03-31

**Authors:** Maliha Ahmed, Tanzy Love, Catherine Moore, Thu H. Le, Jerome Jean-Gilles, Bruce Goldman, Hae Yoon Grace Choung

**Affiliations:** a Department of Medicine, Division of Nephrology, University of Rochester Medical Center, Rochester, NY, USA; b Department of Biostatistics and Computational Biology, University of Rochester Medical Center, Rochester, NY, USA; c Department of Pathology and Laboratory Medicine, Division of Renal Pathology and Electron Microscopy, University of Rochester Medical Center, Rochester, NY, USA

**Keywords:** Lupus-like nephritis, full-house nephropathy, lupus nephritis, autoimmune disease, lupus

## Abstract

**Background:**

A subset of patients without overt systemic lupus erythematosus (SLE) present with biopsy findings typically seen in lupus nephritis (LN). Although a minority eventually develops SLE, many do not. It remains unclear how to classify or treat these patients. Our study attempted to further understand the clinical and pathological characteristics of cases with lupus-like nephritis (LLN).

**Methods:**

Among 2700 native kidney biopsies interpreted at University of Rochester Medical Center (URMC) from 2010 to 2019, we identified 27 patients with biopsies showing lupus-like features (LL-fx) and 96 with LN. Of those with LL-fx, 17 were idiopathic LLN and 10 were associated with a secondary etiology (e.g., infection/drugs).

**Results:**

At the time of biopsy, the LLN-group tended to be slightly older (44 *vs.* 35), male (58.8 *vs.* 17.7%, *p* = .041), and Caucasian (47.0 *vs.* 28.1%, *p* = .005). Chronic kidney disease was the most common biopsy indication in LLN (21.4 *vs.* 2.8%, *p* = .001). Both LN and LLN presented with nephrotic-range proteinuria (mean 5.73 *vs*. 4.40 g/d), and elevated serum creatinine (mean 1.66 *vs*. 1.47 mg/dL). Tubuloreticular inclusions (TRIs; *p* < .001) and fibrous crescents (*p* = .04) were more often seen in LN, while more tubulointerstitial scarring was seen in LLN (*p* = .011). At mean follow-up of 1684 d (range: 31–4323), none of the LLN patients developed ESRD. A subset of both LN and cases with LL-fx overlapped with other autoimmune diseases.

**Conclusions:**

Lupus-like pathologic features are seen in a wide array of disease processes. The findings suggest that LLN may be a manifestation of an autoimmune process that overlaps with SLE.

## Introduction

The diagnosis of lupus nephritis (LN) is strongly suggested by multiple pathological findings including ‘full house’ (FH) immunofluorescence (IF) staining, extra-glomerular immune deposits (EGID), intense C1q staining, endothelial tubuloreticular inclusions (TRIs), and combined mesangial, subendothelial, and subepithelial deposits [1]. However, there are a subset of patients without overt systemic lupus erythematosus (SLE) who present with pathologic features that are indistinguishable from LN. Such cases are encountered occasionally in clinical practice and have been called lupus-like nephritis (LLN), renal-limited LN, or full house nephropathy (FHN). Although a minority of patients eventually develops extra-renal symptoms of SLE, many do not. Moreover, infections, drugs, and other autoimmune diseases have also been associated with renal biopsy findings showing lupus-like features (LL-fx). We performed a single-center study of renal biopsy diagnoses among patients with biopsy findings of LL-fx over a 10-year period. We aimed to explore the range of renal diseases associated with pathologic features of LN in those without clinical evidence of SLE. In addition, we attempted to further understand the clinicopathological characteristics of the subset of cases that were idiopathic, for which we will use the term LLN as used by Huerta et al. [2].

## Materials and methods

In this study, all native kidney biopsies accessioned in University of Rochester Medical Center’s (URMC) Pathology Laboratory from 2010 to 2019 were retrospectively reviewed for evidence of lupus-like (LL) pathology. The following definition of LL-like features (LL-fx) was applied: any combination of 1) FH-positive staining for all Ig and complements with at least 1+ intensity on a 0–3+ scale on IF, 2) TRIs, and 3) EGID with IgG and/or C1q. In addition, we included only patients in the LL-group who had no serologic evidence of SLE and who did not meet Systemic Lupus International Collaborating Clinics Classification (SLICC) or American College of Rheumatology (ACR) SLE classification criteria [3–5]. The LL-group was further divided into idiopathic LLN and those associated with either an identifiable secondary etiology (e.g., infections or drugs) or atypical presentation of another renal disease (e.g., membranous glomerulopathy).

All renal biopsies were processed using standard techniques for light microscopy (LM), IF, and electron microscopy (EM), and interpreted by one of three renal pathologists. Consensus was obtained for each case at weekly conference attended by all three renal pathologists. The following ancillary immunohistochemistry studies were performed on kidney biopsies with membranous glomerulonephritis (MGN) if tissue was available: PLA2R (rabbit polyclonal, Sigma-Aldrich, St. Louis, MO), NELL-1 (rabbit polyclonal, Sigma-Aldrich), THSD7A (rabbit polyclonal, Sigma-Aldrich), EXT1 (rabbit polyclonal, Invitrogen), and EXT2 (rabbit polyclonal, Invitrogen, Waltham, MA).

Clinical information at the time of biopsy and at last follow-up was collected from electronic medical record and referral forms from submitting physicians, if available. Additional information was also obtained from telephone interviews with the referring nephrologist. Clinical data included laboratory parameters, such as serum creatinine, proteinuria, urinalysis, abnormal serologies (such as anti-nuclear antibody (ANA), anti-double-stranded antibody, hepatitis B surface antigen, and hepatitis C antibody, HIV), whether patient satisfied ACR or SLICC criteria for SLE diagnosis in subsequent follow-up, history of infections, other autoimmune diseases, neoplasms, and treatment. The following clinical definitions were used: nephrotic-range proteinuria, UPCR or 24-h urine protein >3.5 g; hypoalbuminemia, serum albumin <3.5 g/dL; hematuria, >5 red blood cells per high power field; ESRD, requiring renal replacement therapy. Focal was defined as involving <50% of glomeruli and diffuse ≥50% of glomeruli. Tubular atrophy and interstitial fibrosis were graded as following: mild, <25%; mild to moderate, 26–35%; moderate, 36–50%; moderate to severe, 51–60%; severe, >60%. Mesangial hypercellularity was defined as >3 mesangial cells per mesangial area; endocapillary hypercellularity, glomerular capillary luminal narrowing or occlusion by increased cells; extracapillary proliferation/crescent, >2 cell layers involving more than 10% of the glomerulus; membranoproliferative pattern, proliferative glomerulonephritis with glomerular basement membrane duplication and interposition of cells and matrix.

To compare continuous values between the LN and LLN groups, we performed two-sample *t*-tests without assuming equal variances. To compare categorical differences, we created cross tabulations of each variable against the group and used chi-squared tests for equal distribution between the LN and LL groups. For all tests, we used a significance level of *α* = 0.05.

## Results

From January 2010 to December 2019, 2700 patients underwent native renal biopsies that were accessioned at URMC. Of these patients, 123 patients with either LN or LL-fx were studied.

## Demographics and clinical presentation

**Figure 1.**
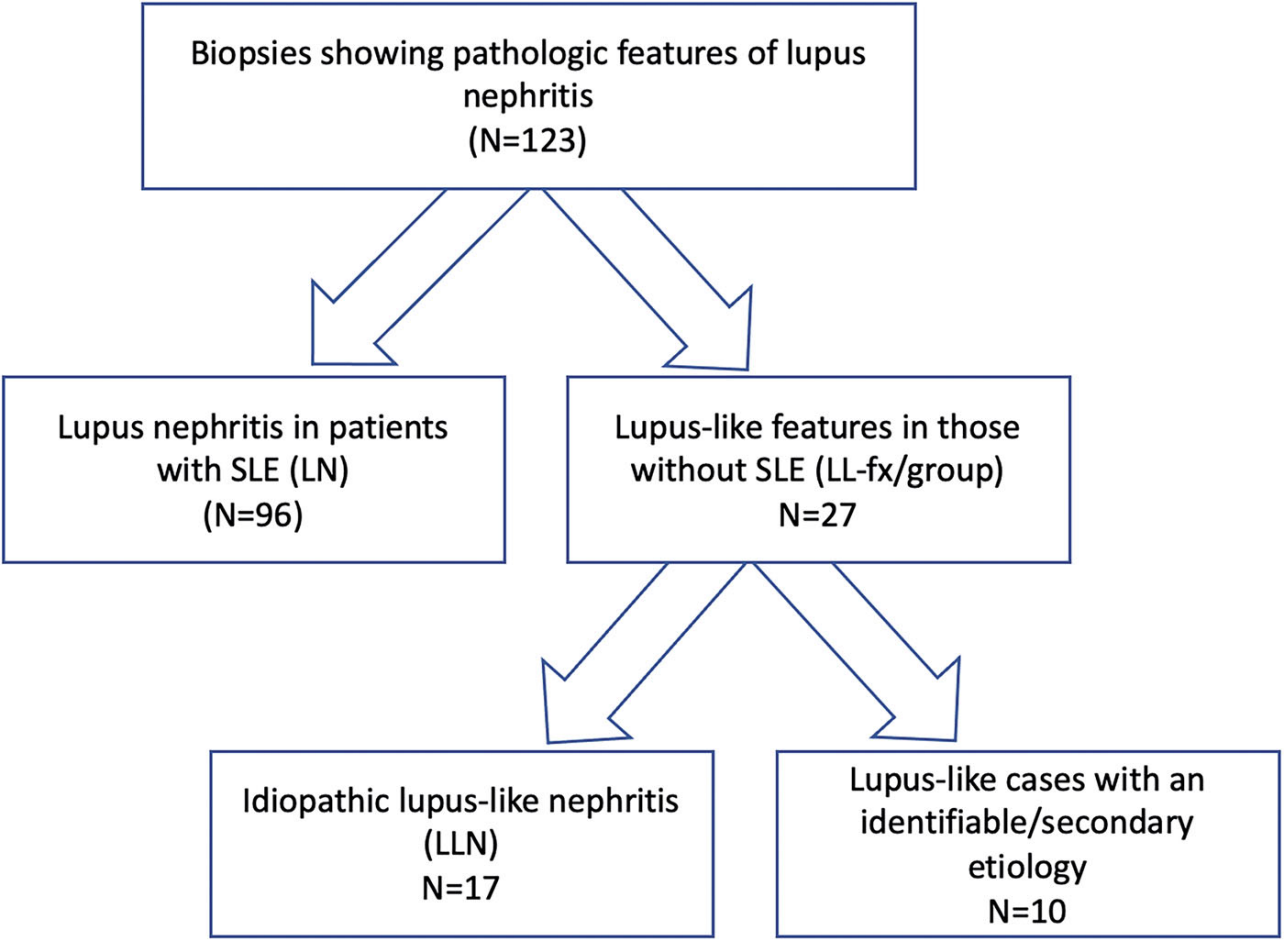
Schematic diagram.

Of the 123 patients with LN or LL-fx, 96 had LN and 27 LL-fx (Figure 1: Schematic). Within the LL-group, 10 had either an associated secondary etiology or an atypical presentation of a renal disease, and 17 were idiopathic LLN. The clinical information between the LN and LLN-groups were compared and are presented in Table 1. All LN patients had a clinical diagnosis of SLE, while none of those with LLN met full criteria for SLE based on ACR or SLICC at the time of biopsy. LN had a mean age of 35 (range: 9–79) with a female to male ratio of 4.6:1, and more often affected Black patients (54%). LLN patients had a slightly higher mean age of 44 (range: 6–87) with reversal of the female to male ratio of 0.7:1 and were more frequently Caucasian (47%). Both groups similarly demonstrated evidence of renal insufficiency, nephrotic-range proteinuria, and hematuria at time of biopsy. ANA and/or anti-double-stranded DNA antibody, and decreased C3 were observed in the majority of LN while absent in LLN (*p* < .001). The most common indication for biopsy in both groups was for proteinuria, while the LLN-group were more often biopsied for CKD (*p* = .041).

**Table 1.** Clinical data at biopsy of patients with lupus *vs.* lupus-like nephritis.

	Mean (range) or *N* (%)		
Clinical features	Lupus nephritis	Lupus-like nephritis	*p* Values
	*N* = 96	*N* = 17	
Age	35.0 (9–79)	44.1 (6–87)	.174
Gender			**.0008**
Male	17 (17.7)	10 (58.8)	–
Female	79 (82.2)	7 (41.1)	–
Race/ethnicity			**.0053**
Caucasian	27 (28.1)	8 (47.0)	–
Black	52 (54.1)	3 (17.6)	–
Hispanic	7 (7.2)	2 (11.7)	–
Asian	6 (6.2)	0 (0.0)	–
Unknown	4 (4.1)	4 (23.5)	–
Serum creatinine (mg/dL)	1.47 (0.45–13.20)	1.66 (0.46–4.50)	.608
Proteinuria (g/d)	4.40 (0.08–27.00)	5.73 (1.00–21.00)	.411
Hematuria	56 (62.2)	9 (52.9)	.654
Meets clinical criteria for SLE^a^	69 (74.1)	0 (0.0)	**<.0001**
Serologies			
Positive ANA and/or dsDNA antibody	88 (97.7)	0 (0.0)	**<.0001**
Low C3	59 (67.0)	2 (14.2)	**.0005**
Low C4	39 (46.9)	3 (21.4)	.135
Positive HBV	1 (1.8)	0 (0.0)	1.000
Positive HCV	1 (1.8)	0 (0.0)	1.000
Positive HIV	0 (0.0)	0 (0.0)	1.000
Indications for biopsy			
Proteinuria	58 (84.0)	13 (86.6)	1.000
Nephrotic syndrome	9 (13.0)	4 (26.6)	.353
Hematuria	17 (24.6)	6 (42.8)	.288
Acute kidney injury	22 (31.8)	4 (28.5)	1.000
Chronic kidney disease	2 (2.8)	3 (21.4)	**.041**

Bold indicates statistically significant values.

^a^Systemic Lupus International Collaborating Clinics classification (SLICC) or American College of Rheumatology (ACR) SLE classification criteria [3–5].

Within the LL-group, eight patients had some form of autoimmune disease other than SLE. Among the idiopathic LLN cohort (Table 2), two had Hashimoto’s thyroiditis (HT), 1 with polymyalgia rheumatica and idiopathic thrombocytopenic purpura (ITP), and 1 with tubulointerstitial nephritis and uveitis (TINU). Two patients had first-degree relatives with SLE and 1 with rheumatoid arthritis (RA). Of the 10 LL patients with a secondary etiology (Table 3), 1 had Crohn’s, 1 with RA, 1 anti-phospholipid syndrome, and 1 psoriasis. Anti-neutrophil cytoplasmic antibody (ANCA)-positive serologies were identified in 3 LL cases (2 anti-PR3 and 1 unknown). Among those with positive ANCAs, 1 had a concurrent HIV and HCV infection. One additional HIV patient had concurrent positive autoimmune serologies for anti-mitochondrial and anti-smooth muscle antibodies and 1 with endocarditis also had positive anti-phospholipid antibody. The LN-group also had other overlapping autoimmune diseases in 10: ITP in 1, sicca syndrome in 1, hemolytic anemia in 1, mixed connective tissue disease (MCTD) in 4 (1 with scleroderma), RA in 3 (1 with also multiple sclerosis), and autoimmune hepatitis (AIH) in 1. Five LN patients had positive ANCAs (1 with double positive for MPO and PR3, 2 with MPO, 1 PR3, and 1 unknown).

**Table 2.** Clinical characteristics and demographics of patients with lupus-like nephritis.

Patient	Age/ gender	Race/ethnicity	Serum creatinine (mg/dL)	Proteinuria (g/d)	Serum albumin	ANA/dsDNA antibody	Complements	Comorbidities	Duration of follow-up (in days)	Treatment	Renal outcome (SCr, mg/dL; proteinuria, g/d)
1	66/M	Caucasian	2.27	1.86	NA	Negative	Low C4	Polymyalgia rheumatica and ITP	4323	Bx#1: lost to f/u Bx#2: initially Cytoxan + steroids, and relapsed then MMF + steroids	SCr 1.1, proteinuria 2.4, Repeat ANA(−)
2	39/F	African American	0.69	1.00	3.80	Negative	Normal	Asthma, pre-DM, hypothyroidism, FH of RA, and ankylosing spondylitis	3044	ACE/ARBs	SCr 0.7, proteinuria 0.7, Repeat ANA(−)
3	87/F	Unknown	1.96	2.30	NA	Negative	Normal	Bipolar disorder	273	MMF and steroids	Deceased SCr 2.5; proteinuria 1.5, Repeat ANA(−)
4	11/F	Hispanic	0.46	1.40	4.20	Negative	Normal	Morbid obesity and pre-DM	2937	ACE/ARBs	SCr 0.8, proteinuria 0.9
5	81/M	Unknown	NA	NA	NA	Negative	Low C3 and C4	HTN		NA	NA
6	14/M	Caucasian	0.70	7.60	1.60	Negative	Low C4 and C4	HTN	2839	Initially steroids + tacrolimus + MMF, No response, and then: Eculizumab + MMF	SCr 1.3, proteinuria 0.4, Repeat ANA (−)
7	48/F	Hispanic	NA	8.0	2.40	Negative	NA	Hashimoto’s thyroiditis, DM, and FH of SLE	3163	MMF and steroids	SCr 1.1, proteinuria 0.0, Repeat ANA(−)
8	32/F	African American	1.04	2.96	3.50	Negative	Normal	Asthma and Eczema	2035	steroids, ACE/ARBs	SCr 0.9, proteinuria 0.1, Repeat ANA(−)
9	18/M	Caucasian	0.54	2.50	3.20	Negative	Normal	Hashimoto’s Thyroiditis and HTN	1874	Tacrolimus, ACE/ARBs	SCr 0.9, proteinuria 2.2, Repeat ANA(−)
10	25/M	Caucasian	0.86	5.70	3.80	Negative	Normal	None	1476	ACE/ARBs	Scr 0.9, proteinuria 0.4
11	59/M	Unknown	2.09	21.00	1.10	Negative	NA	HTN, Asthma, hypothyroidism, and chronic sinus infections	1846	MMF, steroids	SCr 1.1, proteinuria 0.0, Repeat ANA(−)
12	18/M	Caucasian	2.42	1.00	4.60	Negative	Normal	TINU	799	Adalimumab	Scr 1.1, proteinuria 0.0
13	63/M	African American	0.80	10.00	2.80	Negative	Normal	HTN, DM, CVA	317	ACE/ARBs	Scr 0.9, proteinuria 3.1
14	64/M	Unknown	NA	7.00	NA	Negative	NA	HTN, DM	945	ACE/ARBs	Scr 1.6, proteinuria NA
15	57/M	Caucasian	2.80	1.00	2.80	Negative	Normal	HTN, DM, Colon cancer s/p chemorad 20 years ago, Cirrhosis, ?AIH, +ANCA, and + RF	Died soon after Bx	None	Deceased
16	62/F	Caucasian	1.10	17.20	1.60	Negative	Normal	HTN, chronic NSAID use, +ANCA, and FH of SLE	245	Cytoxan, steroids, and rituxan	Scr 1.3, proteinuria 4.5
17	6/F	Caucasian	4.50	1.20	4.30	Negative	Normal	None	809	Steroids	Scr 0.5, proteinuria 0.2, Repeat ANA(−)

ANA: anti-nuclear antibody; ANCA: anti-neutrophil cytoplasmic antibody; BPH: benign prostatic hyperplasia.; Bx: biopsy; CAD: coronary artery disease; COPD: chronic obstructive pulmonary disease; CVA: cerebrovascular accident; DM: diabetes mellitus; dsDNA ab: double-stranded DNA antibody; F: female; HTN: hypertension; M: male; NA: not available; Scr: serum creatinine; SLE: systemic lupus erythematosus; TINU: Tubulointerstitial nephritis and uveitis.

**Table 3.** Clinical characteristics and demographics of lupus-like cases with an identifiable/secondary etiology.

Patient	Age/ gender	Race/ethnicity	Secondary etiology	Serum creatinine (mg/dL)	Proteinuria (g/d)	ANA/dsDNA antibody	Complements	Comorbidities	Duration of follow-up (in days)	Renal outcome (SCr, mg/dL; proteinuria, g/d)
18	68/M	Caucasian	Anti-TNF-α Tx	NA	NA	Positive	Low C3 and C4		NA	NA
19	15/F	Caucasian	Anti-TNF-α Tx	1.14	NA	Positive	Normal	Crohn’s disease	2306	SCr 0.98 and proteinuria 5.2
20	66/M	Caucasian	AVR Endocarditis	3.87	0.52	Negative	Normal	APA ab	29	Deceased
21	73/M	Unknown	anti-PLA2R MGN	NA	NA	NA	NA		NA	NA
22	61/F	Caucasian	Anti-TNF-α Tx	1.79	2.70	Positive	Low C3 and C4	Rheumatoid arthritis	NA	NA
23	66/M	African American	HIV	1.97	2.43	Negative	Normal	HTN, DM, HIV, anti-SMA, and AMA ab	788	SCr 1.9 and proteinuria 2
24	42/M	Other	HIV	2.53	0.52	NA	Low C4	HIV, Hepatitis C, and + ANCA	16	Deceased
25	51/F	Caucasian	anti-PLA2R MGN	0.69	14.00	Negative	Normal	Antiphospholipid antibody syndrome	26	SCr 0.6 and proteinuria 0.6
26	68/M	Caucasian	anti-PLA2R MGN	1.40	7.00	Negative	Normal	Psoriasis and prostate CA	152	Scr 1.1 and proteinuria 2.1
27	65/M	Unknown	anti-PLA2R MGN	3.20	6.00	Negative	NA	HTN and morbid obesity	NA	NA

APA ab: antiphospholipid antibody, AMA: anti-mitochondrial antibodies; ANCA: anti-neutrophil cytoplasmic antibody; anti-SMA: ant-smooth muscle antibody; DM: diabetes mellitus; HTN: hypertension; HIV: human immunodeficiency virus; MGN: membranous glomerulonephritis; M: male; F: female; NA: not available.

## Pathologic features

IF, LM, and EM data for both LN and LLN are presented in Table 4, and more detailed pathologic data for LLN are in Table 5. The majority of both LN and LLN showed FH of immune deposits by IF. Although EGID staining by IgG and/or C1q was approximately three times more frequent in LN compared to LLN, the difference was not statistically significant (*p* = .081). IF intensity (Table 6) in both were not significantly different and showed the strongest positivity for IgG, followed by C3, kappa, lambda, IgM, IgA, and C1q. IgG-subclass staining was performed on 6 LLN cases. The mean intensity for the IgG subclasses revealed from strongest to weakest: IgG1 (1.8+), IgG3 (1.8+), IgG4 (1.5+), and IgG2 (1.0+). The prevalence of TRI by EM was statistically significantly greater in the LN (83.3%) than the LLN-group (29.4%), (*p* < .0001). The differences in SLE class were not statistically significant between LLN and LN (*p* = .093). However, proliferative glomerulonephritis, specifically class-III/IV(±V) (61.4 *vs.* 35.2%), was seen with greater frequency in LN, while non-proliferative class-V (28.1 *vs*. 35.2%) and mesangial proliferative class-II (9.3 *vs.* 23.5%) were more commonly encountered in LLN. All LLN cases with class V only were negative for anti-PLA2R antibody. NELL-1, THSD7A, and EXT1/2 were performed on 4 cases with tissue available for staining, all of which were negative for those markers. Fibrous crescents were more often present in LN (*p* = .04), while LLN cases tended to have more tubulointerstitial scarring (*p* = .011). Two LLN patients had repeat biopsies performed. Patient #1’s second biopsy showed class 4 + 5, which appeared more proliferative than the biopsy from 10 years prior (class 5 only). Both first and second biopsies for patient #6 were class 4, but with more chronic changes in the second biopsy.

**Table 4.** Pathologic data of patients with lupus nephritis *vs.* lupus-like nephritis.

	*N* (%)		
	Lupus nephritis	Lupus-like nephritis	*p* Values
	*N* = 96	*N* = 17	
Immunofluorescence			
Positive extra-glomerular staining	35 (36.8)	2 (11.7)	.081
Electron microscopy			–
Tubuloreticular inclusions	80 (83.3)	5 (29.4)	**<.0001**
Light microscopy			
SLE class			.093
I	1 (1.0)	1 (5.8)	–
II	9 (9.3)	4 (23.5)	–
III/IV ± V	59 (61.4)	6 (35.2)	–
V	27 (28.1)	6 (35.2)	–
Crescents			
Cellular/fibrocellular			.89
<25%	18 (18.7)	3 (17.6)	–
25–50%	5 (5.2)	0 (0.0)	–
>50%	1 (1.0)	1 (5.8)	–
Fibrous			**.04**
<25%	2 (2.0)	0 (0.0)	–
25–50%	1 (1.0)	0 (0.0)	–
>50%	0 (0.0)	0 (0.0)	–
Focal segmental glomerulosclerosis			.993
Focal	26 (27.0)	4 (23.5)	–
Diffuse	0 (0.0)	0 (0.0)	–
Global glomerulosclerosis			.972
Focal	46 (47.9)	8 (47.0)	–
Diffuse	7 (7.2)	1 (5.8)	–
Tubular atrophy and interstitial fibrosis			**.011**
Mild	40 (42.1)	10 (58.8)	–
Mild to moderate	5 (5.2)	2 (11.7)	–
Moderate	11 (11.5)	0 (0.0)	–
Moderate to severe	2 (2.1)	3 (17.6)	–
Severe	6 (6.3)	0 (0.0)	–

Bold indicates statistically significant values.

**Table 5.** Pathological characteristics of patients with lupus-like nephritis.

Patient	Morphologic equivalent for SLE	Proliferative features	Extra-glomerular deposits	Electron microscopy		Crescents		Chronicity		PLA2R	NELL1, THSD7A, EXT1/2
				Deposits	TRI	Cellular/ fibrocellular	Fibrous	Global GS	Tubular atrophy and interstitial fibrosis		
1^a^	Class 5^a^	Mesangial hypercellularity	No	Subepithelial	No	0	0	Focal	None	Negative	NA
2	Class 5	Normocellular	No	Mesangial and subepithelial	Yes	0	0	0	Mild	Negative	NA
3	Class 2	Mesangial hypercellularity	No	Mesangial	No	0	0	Focal	Moderate to severe	–	–
4	Class 2	Mesangial hypercellularity	No	Mesangial	No	0	0	0	Mild	–	–
5	Class 4	Mesangial, endocapillary, and extracapillary hypercellularity	Yes	Mesangial and subendothelial	Yes	<25%	0	Focal	Mild	–	–
6^a^	Class 4^a^	Mesangial, endocapillary, and extracapillary hypercellularity	No	Mesangial, subendothelial, and subepithelial	No	<25%	0	Focal	Mild	–	–
7	Class 2	Mesangial hypercellularity	No	Mesangial	Yes	0	0	0	Mild	–	–
8	Class 1	Normocellular	No	Mesangial	No	0	0	0	Mild	–	–
9	Class 5	Normocellular	No	Subepithelial	Yes	0	0	0	None	Negative	Negative
10	Class 3 + 5	MPGN with mesangial hypercellularity	No	Mesangial, subendothelial, and subepithelial	No	0	0	Focal	Mild to moderate	–	–
11	Class 3 + 5	Mesangial, endocapillary, and extracapillary hypercellularity	No	Subepithelial	No	<25%	0	Focal	Mild	–	–
12	Class 2	Mesangial hypercellularity	No	Mesangial	No	0	0	0	Moderate to severe	–	–
13	Class 5	Mesangial hypercellularity	No	Mesangial, subendothelial, and subepithelial	No	0	0	Focal	Mild to moderate	Negative	Negative
14	Class 5	Normocellular	No	Mesangial and subepithelial	No	0	0	Focal	Moderate to severe	Negative	Negative
15	Class 3	Mesangial and endocapillary hypercellularity	No	Mesangial and subendothelial	No	0	0	0	Mild	–	–
16	Class 5	Mesangial hypercellularity	Yes	Mesangial and subepithelial	No	0	0	Focal	Mild	Negative	Negative
17	Class 4	MPGN with endocapillary and extracapillary hypercellularity	No	Mesangial, subendothelial, and subepithelial	Yes	>50%	0	0	Mild	–	–
Follow-up biopsies											
1 (10 years)	Class 4 + 5	Mesangial, endocapillary, and extracapillary hypercellularity	Yes	Mesangial, subendothelial, and subepithelial	No	<25%	0	Focal	Mild to moderate	–	–
6 (2 years)	Class 4	MPGN with mesangial and endocapillary hypercellularity	Yes	Mesangial and subendothelial	No	0	0	Focal	Mild	–	–

^a^Follow-up biopsies available. All cases showed ‘full house’ staining” except patients 7, 9, 16, and 17.

EM: electron microscopy; NA: not available; GS: glomerulosclerosis; MPGN: membranoproliferative; TRI: tubuloreticular inclusions.

**Table 6.** Immunofluorescence average intensity.

	IgA	IgG	IgM	C3	C1q	Kappa	Lambda
Lupus nephritis *N* = 96	1.2+	2.1+	1.3+	1.9+	1.1+	1.5+	2.0+
Lupus-like nephritis *N* = 17	1.0+	2.5+	1.3+	1.8+	1.0+	1.7+	2.0+

	IgG1	IgG2	IgG3	IgG4
Lupus-like nephritis *N* = 6	1.8+	1.0+	1.8+	1.5+

Of the 27 patients in the LL-group, 10 were ascribed to a secondary etiology or an atypical presentation of a renal disease (Tables 3 and 7). The final diagnosis of six patients was attributed to HIV infection, endocarditis, or anti-TNF-alpha therapy. An additional four cases were positive for anti-PLA2R antibody, the majority of which had mesangial deposits, mesangial hypercellularity, and 1 with endocapillary hypercellularity with membranoproliferative changes (class III + V). Two of the PLA2R-associated MGN cases (Patient 26 and 27) had previous biopsies from outside institutions diagnosed as full-house or lupus-like MGN (LLMN) with 1 showing focal MPGN pattern (Patient 26). Four of these cases had IgG subtyping available; 1 HIV-associated mesangial proliferative GN was IgG1 dominant (2+) with weaker IgG3 and IgG4 (1+); 3 of the cases with anti-PLA2R-antibody-associated MGN were either IgG4 dominant or co-dominant.

**Table 7.** Pathological characteristics of lupus-like cases with an identifiable/secondary etiology^a^.

Patient	Morphologic equivalent for SLE	Proliferative features	Extraglomerular deposits	Electron microscopy		Chronicity	
				Deposits	TRI	Global GS	Tubular atrophy and interstitial fibrosis
18	Class 4	Mesangial, endocapillary, and extracapillary hypercellularity	Yes, TBMs	Mesangial	Yes	Focal	Moderate
19	Class 2	Mesangial hypercellularity	No	Mesangial and subendothelial	No	Focal	Mild to moderate
20	Class 3	Extracapillary hypercellularity	No	Mesangial, subendothelial, and subepithelial	No	Diffuse	Moderate
21	Class 3 + 5	Mesangial and endocapillary hypercellularity	No	Mesangial and subepithelial	No	Focal	Mild
22	Class 4	Mesangial, endocapillary, and extracapillary hypercellularity	Yes, TBMs	Mesangial and subendothelial	No	Focal	Mild to moderate
23	Class 2	Mesangial hypercellularity	No	Mesangial, subendothelial, and subepithelial	No	Focal	Moderate
24	Class 2	Mesangial hypercellularity	No	Mesangial and subepithelial	Yes	Focal	Mild to moderate
25	Class 5	Mesangial hypercellularity	No	Mesangial and subepithelial	No	Focal	Mild
26^b^	Class 5	Mesangial hypercellularity	No	Mesangial and subepithelial	No	None	Mild
27^b^	Class 5	Normocellular	No	Subepithelial	No	Focal	Mild

^a^All cases showed ‘full house’ staining’ except Patients 26 and 27.

^b^Patient 26 and 27 had previous renal biopsies from another institution showing full-house immune staining. Patient 26 had prior biopsy that also showed focal MPGN pattern.

TBMs: tubular basement membranes; TRI: tubuloreticular inclusions; GS: glomerulosclerosis.

## Follow-up evaluation and treatment

Limited follow-up was obtained for 90% with LN and 94% with LLN (Table 8). Average follow-up from time of biopsy for the LN-group was 1732 d (range 3–4068), and for the LLN-group was 1684 d (range: 31–4323). All idiopathic LLN cases with follow-up information available (Table 2) did not develop clinical symptoms of SLE. Repeat ANA was negative in all nine patients who were retested. Of those in the LL-cohort with a secondary etiology (Table 3), three had positive ANAs associated with anti-TNF alpha therapy and were clinically treated as a lupus-like drug reaction.

**Table 8.** Outcomes and follow-up in patients with lupus nephritis and lupus-like nephritis.

	Mean (range) or *N* (%)	
Clinical features	Lupus nephritis	Lupus-like nephritis
	*N* = 96	*N* = 17
Follow-up (in days)	1732 (3–4068)	1684 (31–4323)
Serum creatinine (mg/dL)	1.81 (0.38–20.97)	1.14 (0.52–2.50)
Proteinuria (g/d)	0.80 (0.00–8.62)	1.15 (0.00–4.57)
Hematuria	14 (24.5)	4 (66.6)
ESRD	13 (14.9)	0 (0.0)
Serologies at follow-up		
Low C3	14 (20.5)	0 (0.0)
Low C4	4 (6.0)	1 (16.6)

Out of the patients with follow-up treatment information available, 83% with LN and 64% with LLN received some form of immunosuppression (IS). The most common IS used in LN was mycophenolate mofetil (MMF) (66%), cyclophosphamide (43%), and steroids (41%)) followed by rituximab (29%), hydroxychloroquine (17%), belimumab (11%), and tacrolimus (7%). In LLN, steroids (72%) were used the most, followed by MMF (45%), cyclophosphamide (18%), tacrolimus (18%), rituximab (9%), adalimumab (9%), and eculizumab (9%). Five additional LLN patients were treated with ACE/ARBs only.

Three patients in the LN-group died after suffering from complications of infection and septic shock. Two patients in the LLN-group died, 1 from a cause unrelated to the renal disease and the other had an accelerated decline in health soon after biopsy thought to be related to possible AIH or lymphoma. Thirteen patients with LN (14.9%) and none of the LLN developed ESRD. Most of the LN cases (76%) with ESRD had class III/IV(±V).

## Discussion/conclusion

**Figures 2.**
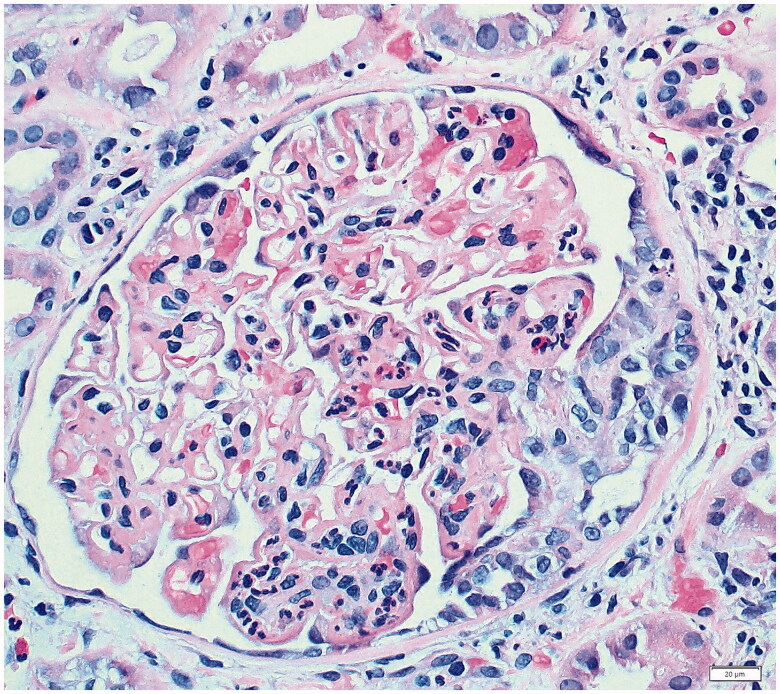
Light microscopy (H&E, 400x): Endocapillary and extracapillary proliferative glomerulonephritis in a LLN case.

**Figure 3.**
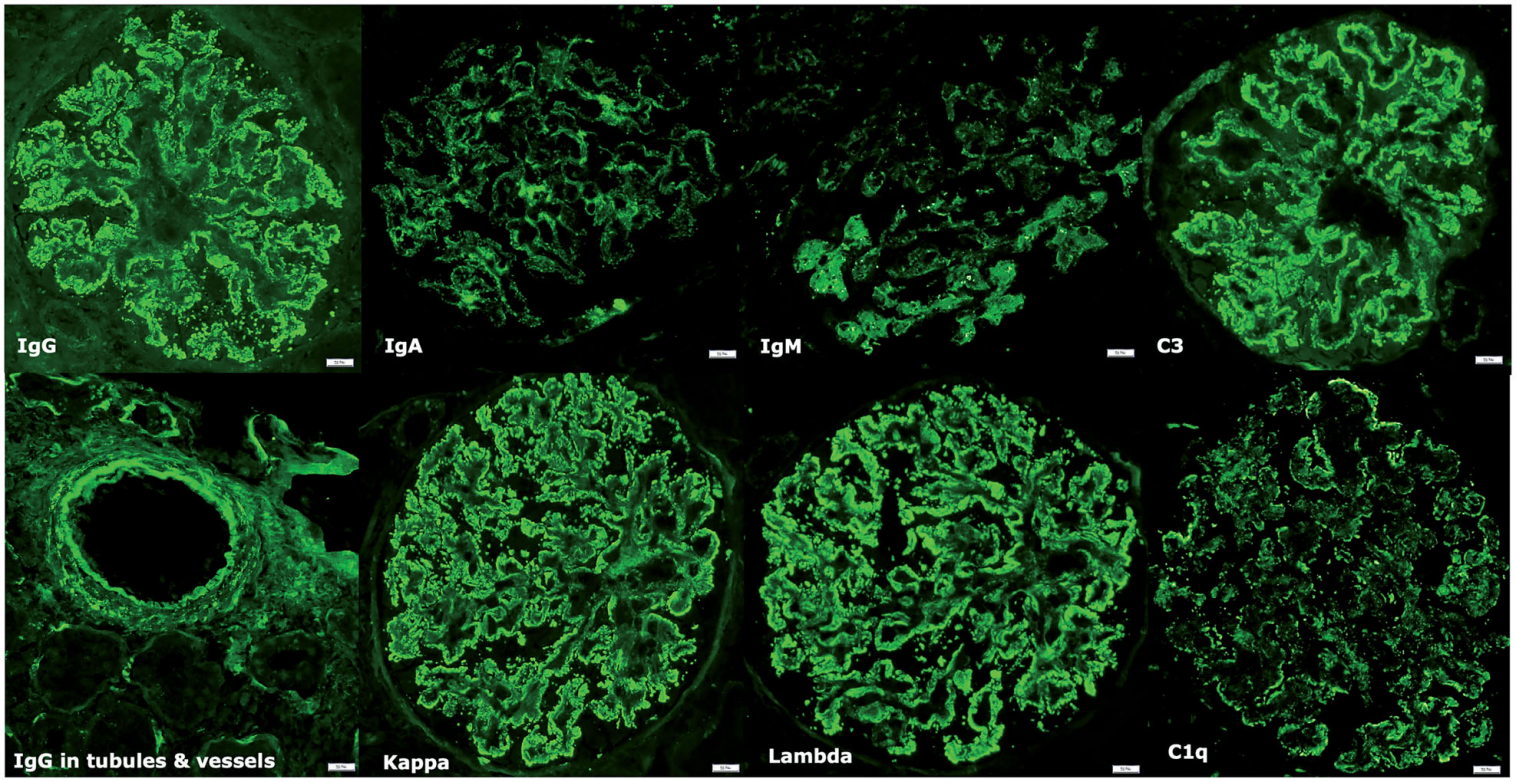
Immunofluorescence microscopy: ‘Full house’ immune deposition by immunofluorescence in a LLN case. Lower right hand panel shows IgG staining within the tubular basement membranes and vessel walls.

Herein, we report a cohort of patients presenting with biopsy findings exhibiting LL-fx. Unlike other studies where the main criteria of FH immune deposition by IF were used, our study focused on including cases that had a combination of any of the following features that are typically seen in LN (Figures 2 and 3); that is, IgG-dominance, FH immune staining of all immunoglobulins and complement, EGID involving vessels and tubular basement membranes by IgG and C1q, intense C1q staining, and ultrastructural findings of endothelial TRIs and presence of mesangial, subendothelial, and/or subepithelial deposits [1,2]. Because the inclusion criteria for our cohort were not restricted to cases with FH staining, we choose to use the term LLN rather than FHN.

Similar to previous studies of FHN [6,7], our idiopathic LLN cohort showed several comparable pathologic findings; namely, the presence of more Class V and Class II morphologic changes compared to proliferative lesions (e.g., class III, IV, and V + III/IV), while the LN cohort had notably more frequent class III/IV(±V). Although the presence of certain chronic lesions (e.g., global and segmental glomerulosclerosis) were comparable in both groups, unlike prior studies, the LLN cohort showed slightly more tubulointerstitial scarring, and significantly less fibrous crescents than the LN group. Cases of FHN typically present with heavier proteinuria (esp. nephrotic syndrome) and less hypocomplementemia compared to those with LN and are male predominant (*vs.* female predominant in LN). Our LLN cohort showed similar findings at initial presentation but with slightly worse renal function. Moreover, LLN was identified with more frequency in Caucasians than in Blacks, Hispanics, and Asians. Of those with follow-up information available, none of the patients with LLN progressed to ESRD whereas a minority of those with LN did. This is in contrast with several case series and studies showing worse prognosis in FHN compared to LN [2,7,8]. Rijnink et al. found in their cohort of FHN with class III/IV(±V) more rapid progression to ESRD and death. These cases progressed to ESRD despite IS, though cytotoxic IS was not used in their cohort. Similarly, another study found worse prognosis in the LLMN cases than lupus membranous nephritis (LMN) but better than primary/idiopathic MGN [2,7]. We found the converse in our cohort of class III/IV(±V), which showed increased incidence of ESRD in those with LN compared to LLN. Instead, our findings appear to parallel those of a study of FHN in pediatric patients, most of whom appear to have either partial or complete remission [9]. In that study, the authors attributed the better clinical course compared to those with SLE as a result of aggressive induction therapy. In our cohort of LLN who had class III/IV(±V) and treatment information available, more than half of the patients received IS including steroids and cytotoxic drugs. With that said, the LN group with III/IV(±V) had slightly more fibrous crescents and moderate to severe tubulointerstitial scarring compared to the LLN cohort with similar class (3 *vs.* 0% and 17 *vs*. 0%, respectively), suggesting that the comparatively less tubulointerstitial scarring and fibrous crescents may account for at least in part to the better clinical course in our LLN group. We found no significant difference in outcome between those with LLN who received IS and those who either did not receive any treatment or were given ACE/ARBs only. This may be due to small sample size and the fact that those who received ACE/ARBs only or no treatment did not have significantly active proliferative glomerulonephritis or chronicity.

Some authors propose that FHN or LLN may represent a form of latent SLE due to the observation that a small number of cases present with clinical and/or serologic evidence of LN several months to years later [10]. Yet, none of the idiopathic FHN cases in Rijnink et al. [7,11] study developed SLE even at 20 years follow-up and no other etiology was found. Similar cases have been reported by Huerta et al. [2], in which none of the patients developed clinical or serologic evidence of SLE at follow-up. Similarly, none of our cases developed SLE during follow-up of up to 11 years. Smith et al. [12] found that a subset of patients presenting with organ damage including biopsy of class III/IV was ANA-negative with half eventually developing ANA-positivity. The authors speculate that ANA-negativity, particularly in young SLE patients, may be related to genetic factors that contribute to the inflammation and tissue damage in the absence of autoantibodies.

Renal involvement in SLE is often favored based on a combination of several pathological features characteristic of LN on renal biopsy and supported in the context of serological and systemic symptoms of SLE. However, similar pathologic findings are occasionally encountered in patients without clinical evidence of SLE. For example, FH glomerular staining pattern is one of several pathologic features that, although highly characteristic of LN, is not specific to it and can occur as an atypical pathologic presentation in various other entities including membranous nephropathy (MGN), IgA nephropathy (IgAN), ANCA-associated glomerulonephritis, C1q nephropathy, among others [6,13,14]. C1q deposition has been described in rare cases of IgAN and has been associated with worse renal outcome and severe pathologic features [15]. Infectious and drug-related processes may present with a full-house or lupus-like pathologic features. LL-fx have been reported in COVID, HIV, bacterial endocarditis, and HBV [14,16–18]. Drug-related reactions, such as immune-check point inhibitors, hydralazine, sulfasalazine, procainamide, TNF-α inhibitors, and multikinase inhibitor regorafenib have been described [19–23]. Features of LN (particularly with extra-glomerular deposits involving tubular basement membranes) can also be encountered in other autoimmune diseases, such as MCTD, Sjogren syndrome, hypocomplementemic urticarial vasculitis syndrome, IgG4 related nephritis, and RA [24]. Endothelial TRIs, which have also been commonly associated with SLE, can also be encountered in the setting of elevated interferons, such as related to viral infection (notably HIV and COVID19) and interferon therapy in Hepatitis.

Because of the strong pathologic similarities with LN, some speculate that both LN and LLN share similar patho-mechanisms. Autoantibody formation and resultant inflammation in SLE has been hypothesized to be related to dysregulated apoptosis and poor clearance of apoptotic debris, which subsequently exposes cryptic self-antigens to an abnormal immune response leading to self-directed antibodies and defective clearance of immune complexes [25]. Some authors have suggested that the full-house immune deposition seen in LN may be due to the pronounced polyclonal B-cell activation and immune response in the setting of these mechanisms and that LLN could very well be a manifestation of a similar aberrant process [7]. This process may also explain why some cases with delayed SLE presentations are ANA-negative initially. Auto-antibodies directed to other components of nuclear material, such as nucleosomes or DNA-histone complex have been proposed as potential antigenic factors LN [26]. Autoantibodies to nucleosomes have been found to either bind to deposited nucleosomes or cross-react with glomerular constituents, and its formation appears to precede anti-dsDNA or anti-histone autoantibodies [27,28]. Thus tests for specific autoantibodies against a particular nuclear component may be positive while ANA is negative [29]. Furthermore, it is conceivable that some LLN patients who do not present with serological evidence of SLE even after many years of follow-up may have an undetectable autoantibody to a specific component of nuclear material for which a test is unavailable.

Moreover, cases secondary to infections and drugs have also been linked to increased autoimmunity. COVID19 infections triggering autoimmune diseases have been documented, such as anti-glomerular basement membrane disease, IgA vasculitis, and ANCA vasculitis [30–32]. Endocarditis-associated GN has been associated with multiple autoimmune markers including ANCA and ANA serologies [33,34]. ANCA has been reported in various infections including viral (such as Hepatitis B and C), bacterial, fungal, parasitic, and chronic infections [35]. Drugs are also well known to cause lupus-like reactions and are often encountered with multiple positive autoimmune serologies [36–38]. For example, hydralazine-associated ANCA often presents with dual ANCA positivity with ANA and anti-histone antibodies. Further confounding this is that some patients with infections and drug reactions present with rheumatological symptoms [39,40]. There is growing evidence to suggest that exogenous antigens trigger an autoimmune reaction in genetically susceptible individuals as a ‘second hit’ through multiple mechanisms including a dysregulated immune system, molecular mimicry, and infection-induced changes in epitope conformation (e.g., unmasking of cryptic antigens) [41].

In addition to potential triggers of autoimmune disease, a subset of LN and LL cases in our study had other preexisting autoimmune diseases, such as MCTD and RA, which has not been described in previous studies. Notably, a small subset of the LLN group had thyroid disease including HT. Autoimmune thyroiditis, such as HT and other forms of thyroid disease have been described in association with SLE, with 14–51% of SLE patient reported to have thyroid autoantibodies [42–45]. HT has also been associated with glomerular diseases, the most common of which were FSGS, MGN, MCD, chronic glomerulonephritis (GN), IgAN, and amyloidosis; though GN with LL-features has not been reported [46,47]. In other case series and reports, some of the LLN patients developed SLE later on, which we did not observe. Nonetheless, these observations seem to suggest that LLN is a manifestation of an autoimmune process that has overlaps with SLE and that multiple factors including drugs and infections may be a trigger in those with a genetic predisposition.

Given so much overlap between SLE and multiple other entities, it is difficult to diagnose LN on pathology alone. Yet, one of the requirements for the diagnosis of SLE using the SLICC criteria includes LN as a sole clinical criterion in the presence of positive ANA or anti-ds DNA antibodies [5]. Even the most recent EULAR/ACR criteria places heavy weight on biopsy findings as part of the diagnostic criteria for SLE [48]. This is problematic because the pathologic diagnosis of LN requires the presence of SLE [24]. There is no specific definition or criteria set for the diagnosis of LN. Moreover, the International Society of Nephrology/Renal Pathology Society (ISN-RPS) Working Group on the Classification of LN states that renal biopsy findings cannot be used to establish a diagnosis of SLE [49]. Indeed, Kudose et al. investigated the combination of five pathologic features to address this conundrum (such as ‘full house’ staining by IF, strong C1q staining, presence of extraglomerular deposits, subendothelial and subepithelial deposits, and endothelial TRIs), and found the presence of at least 2–5 of the criteria allowed for increasing specificity from 0.89 to 0.98 with sensitivity of 0.89–0.66 for the diagnosis of LN [1]. Given the high specificity and acceptable sensitivity, this criteria set may serve as a potential basis for the pathologic diagnosis of LN as part of SLICC and EULAR/ACR criteria. Nonetheless, the authors in that study have also identified rare cases with 3 or more of the 5 criteria in the setting of non-lupus GN, emphasizing that the pathology requires evaluation in tandem with clinical and laboratory data.

Our study has several limitations owing to its retrospective nature including small sample size, varying treatment regimens, lack of available clinical and laboratory data at presentation and follow-up, and inconsistent follow-up periods. Pathologic studies, such as NELL1, EXT1/2, THSD7A, and IgG subclass analysis were not available in some of the cases.

In conclusion, LLN demonstrates biopsy findings similar to LN in those who do not exhibit clinical evidence of SLE, but with less proliferative changes and slightly more tubulointerstitial scarring. In our cohort, these patients were often male, Caucasian, and were more likely to present with nephrotic proteinuria (particularly nephrotic syndrome) and CKD. However, they do not appear to have worse prognosis than those with LN. Pathologic features of LN are seen in a wide array of disease processes, all of which may be related to underlying predisposition to autoimmunity. LLN may be a manifestation of a complex autoimmune process overlapping with SLE and there are multiple possible etiologic triggers including other underlying autoimmune diseases (such as RA, MCTD, among others), infection, and drugs.

## Statement of ethics

This research was performed under approval of an institutional review board (STUDY00005141) and all ethical principles and guidelines for the protection of human subjects were followed.

## Abbreviations

AIH: autoimmune hepatitis
ANA: anti-nuclear antibody
ANCA: anti-neutrophil cytoplasmic antibody
EGID: extra-glomerular immune deposits
EM: electron microscopy
FH: full house
FHN: full house nephropathy
HT: Hashimoto's thyroiditis
IF: immunofluorescence
IS: immunosuppression
ITP: idiopathic thrombocytopenic purpura
LL: lupus-like
LL-fx: lupus-like features
LM: light microscopy
LMN: lupus membranous nephritis
LN: lupus nephritis
LLN: lupus-like nephritis
MCTD: mixed connective tissue disease
MGN: membranous nephropathy
RA: rheumatoid arthritis
SLE: systemic lupus erythematosus
TINU: tubulointerstitial nephritis and uveitis
TRI: tubuloreticular inclusions
